# Autophagic cell death participates in POMC-induced melanoma suppression

**DOI:** 10.1038/s41420-018-0070-5

**Published:** 2018-07-10

**Authors:** Jian-Ching Wu, Han-En Tsai, Guei-Sheung Liu, Chieh-Shan Wu, Ming-Hong Tai

**Affiliations:** 1 Doctoral Degree Program in Marine Biotechnology, National Sun Yat-sen University, 70 Lien-Hai Road, Kaohsiung, 80424 Taiwan; 2 Doctoral Degree Program in Marine Biotechnology, Academia Sinica, 128 Academia Road, Section 2, Nankang, Taipei, 11529 Taiwan; 3 Institute of Chemistry, Academia Sinica, 128 Academia Road, Section 2, Nankang, Taipei, 11529 Taiwan; 4 Centre for Eye Research Australia, Royal Victorian Eye and Ear Hospital, East Melbourne, VIC Australia; 5 Ophthalmology, Department of Surgery, University of Melbourne, East Melbourne, VIC Australia; 6 Department of Dermatology, Kaohsiung Veterans General Hospital, Kaohsiung, 813 Taiwan; 7 Institute of Biomedical Science, National Sun Yat-sen University, Kaohsiung, 804 Taiwan; 8 Center for Neuroscience, National Sun Yat-sen University, Kaohsiung, 804 Taiwan

**Keywords:** Cancer microenvironment, Apoptosis

## Abstract

Hypoxia in tumors is known to trigger the pro-survival pathways such as autophagy. Systemic proopiomelanocortin (POMC) gene therapy suppresses melanoma through apoptosis induction and neovascularization blockage. In this study, we investigated the crosstalk between autophagic and apoptotic signaling in POMC-mediated melanoma suppression. By histological and immunoblot analysis, it was shown that POMC-treated melanoma tissues exhibited the prominent LC3 immunostaining, which was correlated with reduced CD31-positive tumor vascularization. Such autophagy induction could be recapitulated in melanoma cells receiving POMC gene delivery and hypoxia-mimicking agent cobalt chloride (CoCl_2_). We then utilized the POMC-derived peptide α-MSH with CoCl_2_ to elicit the autophagy as well as apoptosis in cultured melanoma cells. To delineate the role of autophagy during cell death, application of autophagy-inducer rapamycin enhanced, whereas autophagy inhibitor 3-MA attenuated, the α-MSH-induced apoptosis in melanoma cells. Genetic silencing of ATG5, an autophagy regulator, by RNA interference perturbed the α-MSH-induced apoptosis in melanoma cells. Finally, it was delineated that α-MSH stimulated the HIF-1α signaling as well as the expression of BNIP3/BNIP3L, thereby promoting the autophagy and apoptosis in melanoma cells. Therefore, the present study unveiled a unique function of autophagy in promoting cell death during POMC-mediated melanoma suppression via α-MSH/HIF-1α/BNIP3/BNIP3L signaling pathway.

## Introduction

Hypoxia is a common characteristic of pathological features presenting in solid tumors and is associated with a poor outcome. Generally, tumor cells are well adapted to moderate hypoxia by inducing several genes involved in angiogenesis, glycolysis, glucose uptake and metastasis^1^. However, in complex conditions such as glucose deprivation or acidosis, hypoxia is also capable of inducing apoptosis and autophagic cell death through a hypoxia-inducible factor-1 (HIF-1)-independent manner or reactive oxygen species (ROS) stimuli^2–5^. Recent studies have shown that hypoxia-induced autophagy and apoptosis are crosstalk from many common upstream pathways, indicating that they can regulate each other^6,7^. Although the relationship between autophagy and apoptosis has been known for rather complex during hypoxia, the role of autophagy and molecular regulatory mechanisms between autophagy and apoptosis are not clearly understood.

Autophagy is a self-degradative process for the cellular stress adaptation response that maintains cell homeostasis and protection^8^. Once activated, the autophagic process initially requires the dissociated Beclin-1 from Bcl-2 or Bcl-X_L_ binds to class ΙΙΙ phosphatidylinositol 3-kinase (PIK3C3 or Vps34) that forms an initiation complex and recruits autophagy-related protein 7 (ATG7) to the developing phagopore. Autophagosomal elongation then recruits two ubiquitin-like conjugation systems, ATG12-ATG5 and subsequent phosphatidylethanolamine conjugated form of the microtubule-associated protein light chain 3 (LC3-ΙΙ)^9^. Autophagy not only protects against diverse pathologies, such as infections, neurodegeneration, aging, and inflammation^10,11^ but also modulates CD4^+^ T cell population and enhancement of adaptive immune responses^12^. Moreover, accumulating studies point to the dual role of autophagy in tumor microenvironment: one aspect is on maintaining tumor cells survival and contributing to tumor progression, and the other one is reversely to promote cancer cell death in some cases^13–16^. For example, when tumor cells exposed to double stresses such as hypoxia and ATP deprivation situation, inducible autophagy can lead to the mitochondrial dysfunction and cell death by HIF-1α-mediated Bcl-2 gene families, such as *BNIP3* (Bcl-2 adenovirus E1a nineteen kilodalton interacting protein 3) and *BNIP3L* (Bcl-2 adenovirus E1a nineteen kilodalton interacting protein 3-like)^17–19^. Therefore, targeting autophagy for cancer therapy may present selectivity dependent on cell adaptation in tumor microenvironment.

Proopiomelanocortin (POMC) is a precursor of multiple peptide hormones, which is expressed in hypothalamic neurons and melanocytes and keratinocytes. POMC products (the adrenocorticotrophic hormone, melanocyte-stimulating hormones [MSHs], and β-endorphin) manage pleiotropic functions, including pigmentation, adrenocortical function, regulation of energy homeostasis, and immunity modulation^20–23^. Our previous studies showed that utilizing the POMC gene carrying by adenovirus is efficient way for Lewis lung carcinoma and melanoma suppression in vivo, we then characterized that POMC-derived peptide α-MSH not only inhibits the colony-forming capacity and invasion of melanoma cells^24–26^ but also retards the tube formation and migration in endothelial cells^27,28^. In addition, α-MSH can mimic POMC-induced apoptosis during hypoxia by increasing ROS generation^29^. However,  the mechanisms of POMC-induced apoptosis in melanoma cells under hypoxic condition is still notenough comprehension. This study aimed to reveal how autophagy and apoptosis work together in response to double stress (hypoxia and α-MSH) stimuli in melanoma cells.

## Results

### POMC gene therapy elicits autophagy in melanoma in vivo

**Fig. 1:**
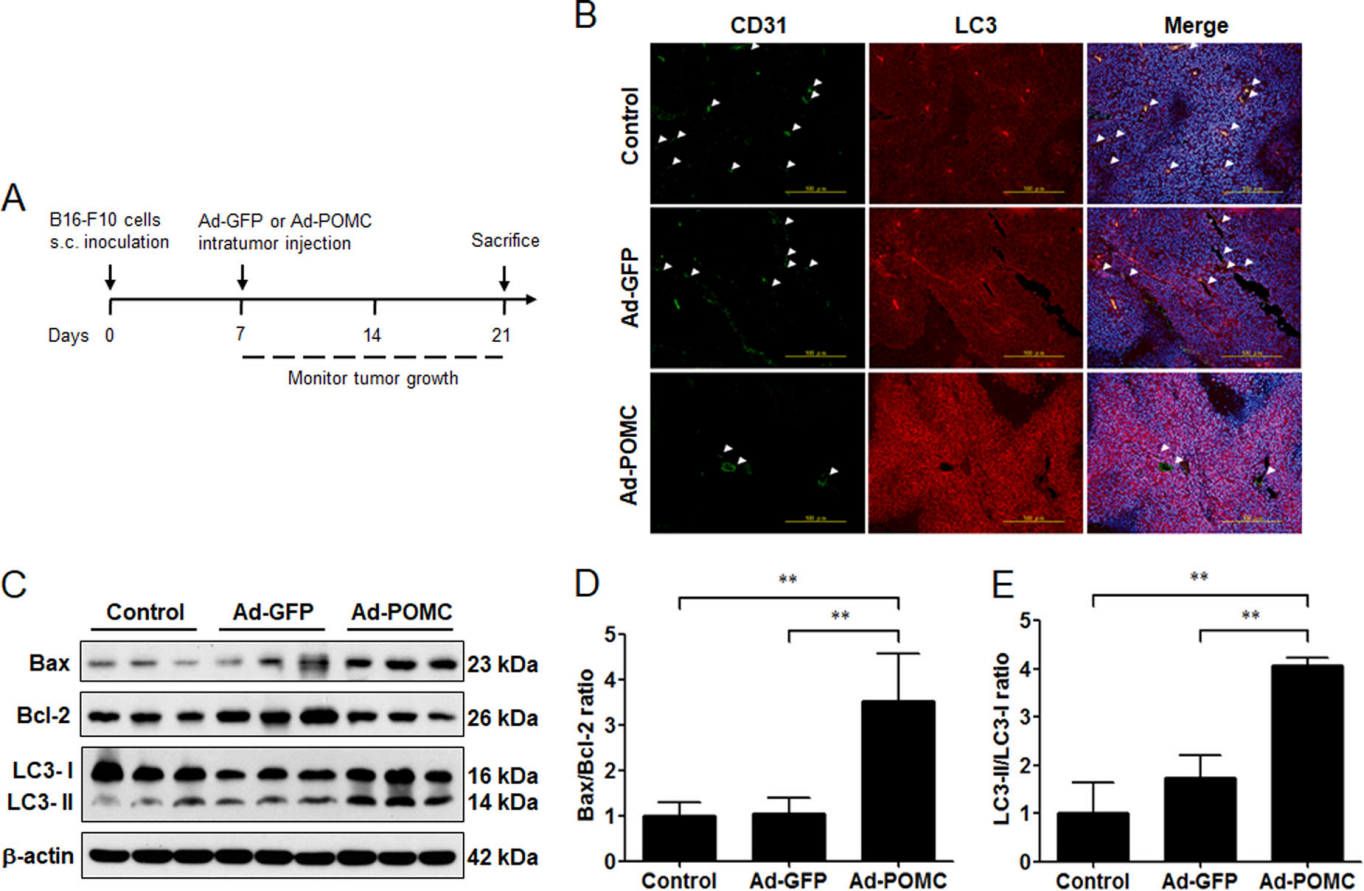
Elevated autophagic activity in Ad-POMC-treated melanoma. **a** After subcutaneous inoculation of B16-F10 cells in C57BL/6 mice for 7 days (5 × 10^5^ cells in 0.1 ml of PBS per mice), mice were administered adenovirus vector carrying GFP or POMC gene (1 × 10^9^ pfu in 0.1 of PBS) by intratumor injection. The melanoma tissues were dissected for the following experimental analysis at the indicated end point. **b** Autophagic LC3 (red) and CD31 (green) co-staining in Ad-POMC-treated melanoma tissues. The number of CD31-positive microvessel was reduced in Ad-POMC-treated melanoma tissues (white arrowheads). Cell nucleus was stained by DAPI (blue). Scale bar, 500 µm. **c** Immunoblot analysis of LC3 expression in melanoma tissues. The protein extracts from different groups of melanoma tissues (*n* = 3 per group) were analyzed by immunoblot analysis. β-Actin was used as internal control. **d**, **e** The ratio of Bax/Bcl-2 and LC3-ΙΙ/LC3-Ι was quantified by relative intensities of protein bands. **p < 0.01. Thus POMC gene therapy elevated autophagy and Bax/Bcl-2 ratio in melanoma tissues

Because it is known that POMC gene therapy induces apoptosis in melanoma via α-MSH during hypoxic challenge^29^, we would like to elucidate whether autophagy also occurred and participated in POMC-induced melanoma suppression. To address this question, after executing the primary melanoma model as shown in Fig. 1a, the tumor tissues were fixed and sectioned for histological analysis. By immunofluorescence staining, it was revealed that a strong LC3 staining was observed in adenovirus (Ad)-POMC-infected tumors compared with Ad-green fluorescent protein (GFP)-infected tumors, and it was correlated with low density of CD31-positive microvessels, implying that POMC-induced autophagy might occur under hypoxic stress (Fig. 1b). Moreover, immunoblot analysis revealed that the protein levels of Bax and LC3-ΙΙ in Ad-POMC-infected tumor tissues were higher than that in Ad-GFP-infected group (Fig. 1c). Quantification analysis further indicated that the Bax/Bcl-2 ratio and LC3-ΙΙ/LC3-Ι conversion were significantly increased in Ad-POMC-infected melanoma tissues (Fig. 1d, e). These results indicated that POMC gene therapy induces the autophagic flux in melanoma.

### POMC gene delivery promotes the autophagic formation in B16-F10 melanoma cells during hypoxia

**Fig. 2:**
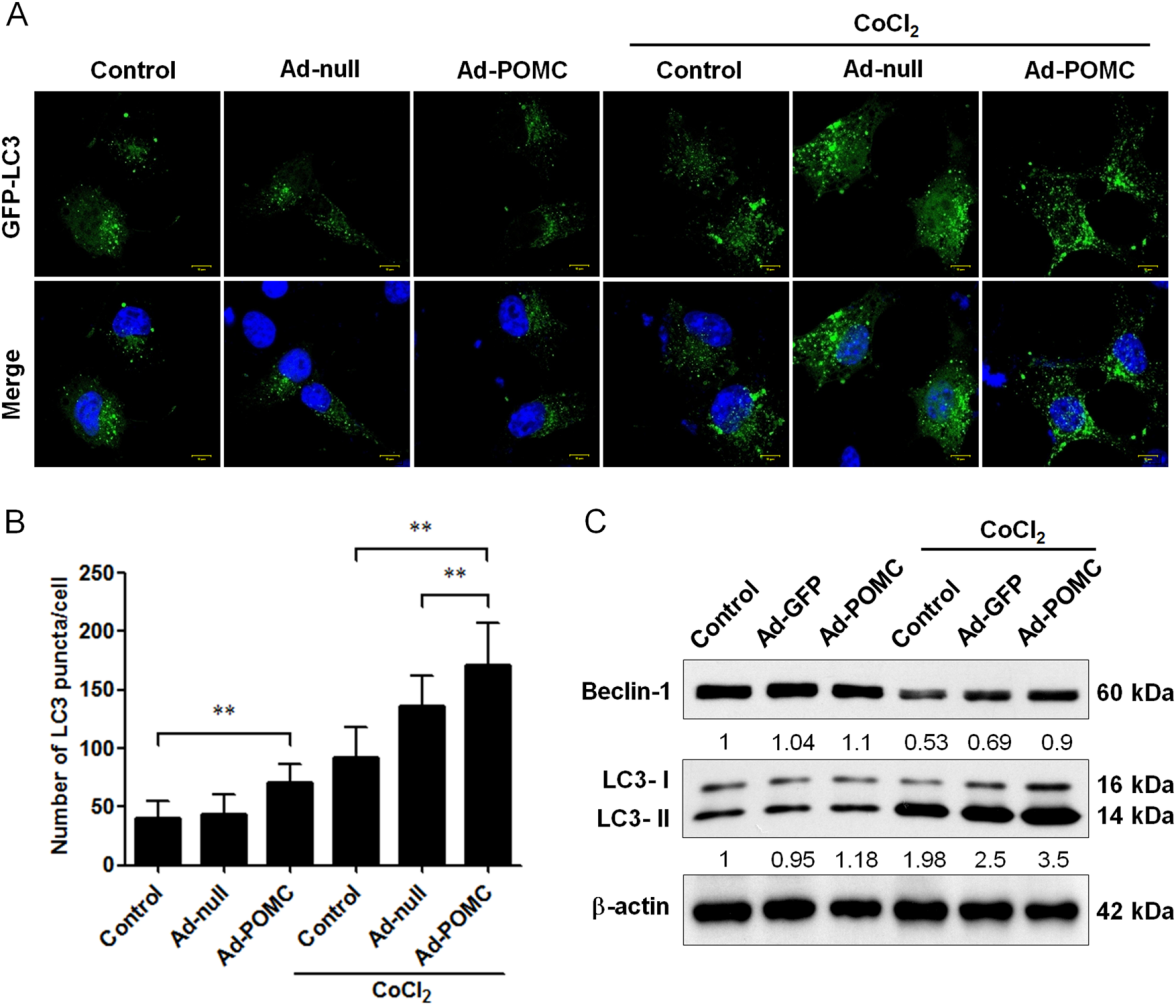
Effect of POMC gene delivery on autophagy in melanoma cells during hypoxia. **a** After transfection with GFP-LC3 plasmid for 24 h, cells were infected with adenovirus vectors at MOI of 1000 for 24 h and then incubated in the presence or absence of CoCl_2_ (100 μM) for another 24 h. POMC gene delivery increased GFP-LC3 spots in cells exposed to hypoxic condition. **b** The number of GFP-LC3 spots was monitored by confocal microscopy and quantified. Scale bar, 10 µm. **c** Cell lysates were analyzed by immunoblot with the indicated antibodies. β-Actin was used as an internal control for loading and transfer. **p < 0.01. Hence, POMC gene delivery enhanced autophagic activities in B16-F10 melanoma cells during hypoxia

**Fig. 3:**
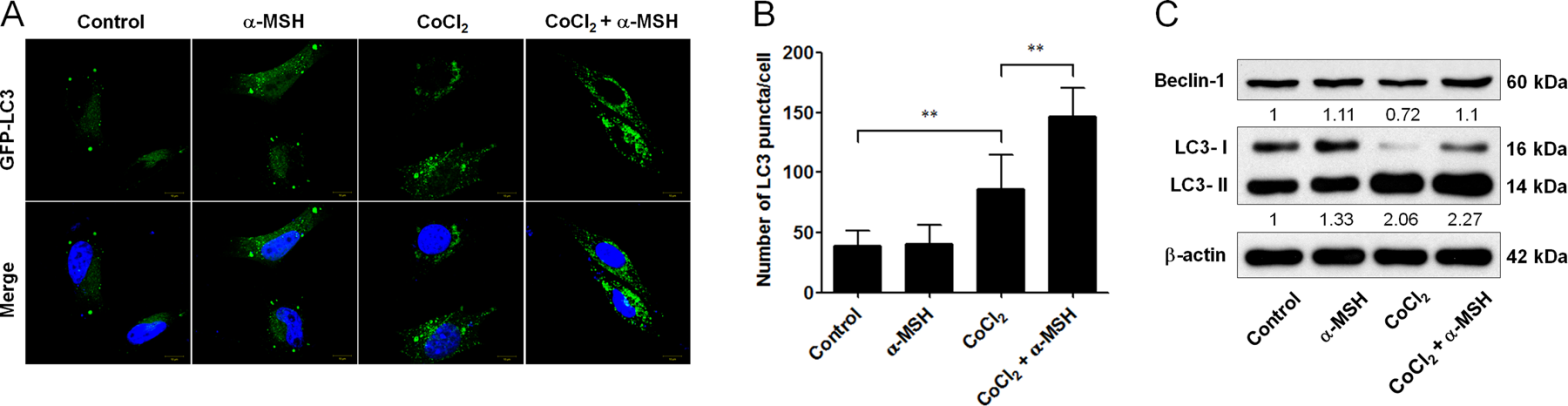
Effect of α-MSH on autophagy in B16-F10 melanoma cells during hypoxia. **a** α-MSH increased GFP-LC3 spots in cells exposed to hypoxic condition. **b** The number of GFP-LC3 spots was observed by confocal microscopy and quantified. Scale bar, 10 µm. **c** α-MSH enhanced the protein level of Beclin 1 and LC3-ΙΙ in B16-F10 melanoma cells during hypoxia. **p < 0.01. α-MSH enhanced autophagic activities in B16-F10 melanoma cells during hypoxia

Subsequently, we investigated whether POMC gene delivery could enhance the autophagy in B16-F10 melanoma cells. By GFP-LC3 puncta formation assay, it was found that POMC gene delivery marginally promoted the GFP-LC3 puncta spots in melanoma cells under normoxic condition (Fig. 2a, b). Strikingly, POMC gene delivery significantly stimulated the GFP-LC3 puncta formation in melanoma cells during hypoxia by cobalt chloride (CoCl_2_; Fig. 2a, b). To confirm this finding, immunoblot analysis showed that POMC gene delivery reversed the hypoxia-induced Beclin 1 downregulation and increased LC3-I and -ΙΙ protein levels under hypoxic challenge, although it did not affect the LC3-ΙΙ/LC3-Ι conversion, suggesting that POMC gene delivery not only stimulated the autophagic activity but also enhanced the total protein level of LC3 (Fig. 3c). These results suggested that autophagy could be recapitulated in B16-F10 melanoma cells by combined POMC overexpression with hypoxia-inducing agent.

### α-MSH evoked the autophagy in B16-F10 melanoma cells during hypoxia

α-MSH has been demonstrated as the primary neuropeptide responsible for the apoptosis-inducing as well as the anti-neoplastic function of POMC gene therapy in melanoma^24,27,29^. Thus we examined whether α-MSH application also modulated the autophagy in melanoma cells. By using GFP-LC3 puncta formation assay, it was shown that α-MSH prominently increased the GFP-LC3 puncta spots in CoCl_2_-treated B16-F10 melanoma cells (Fig. 3a, b) but had no effect on autophagy in normoxic condition. Consistently, immunoblot analysis revealed that α-MSH enhanced LC3-ΙΙ protein expression and reversed hypoxia-induced Beclin 1 suppression during hypoxia (Fig. 3c). These findings suggested that α-MSH was sufficient to mimic the POMC-mediated autophagy regulation in melanoma cells.

### Autophagy inhibitor 3-methyladenine (3-MA) suppresses, while autophagy-inducer rapamycin enhances, the α-MSH-induced apoptosis of melanoma cells during hypoxia

**Fig. 4:**
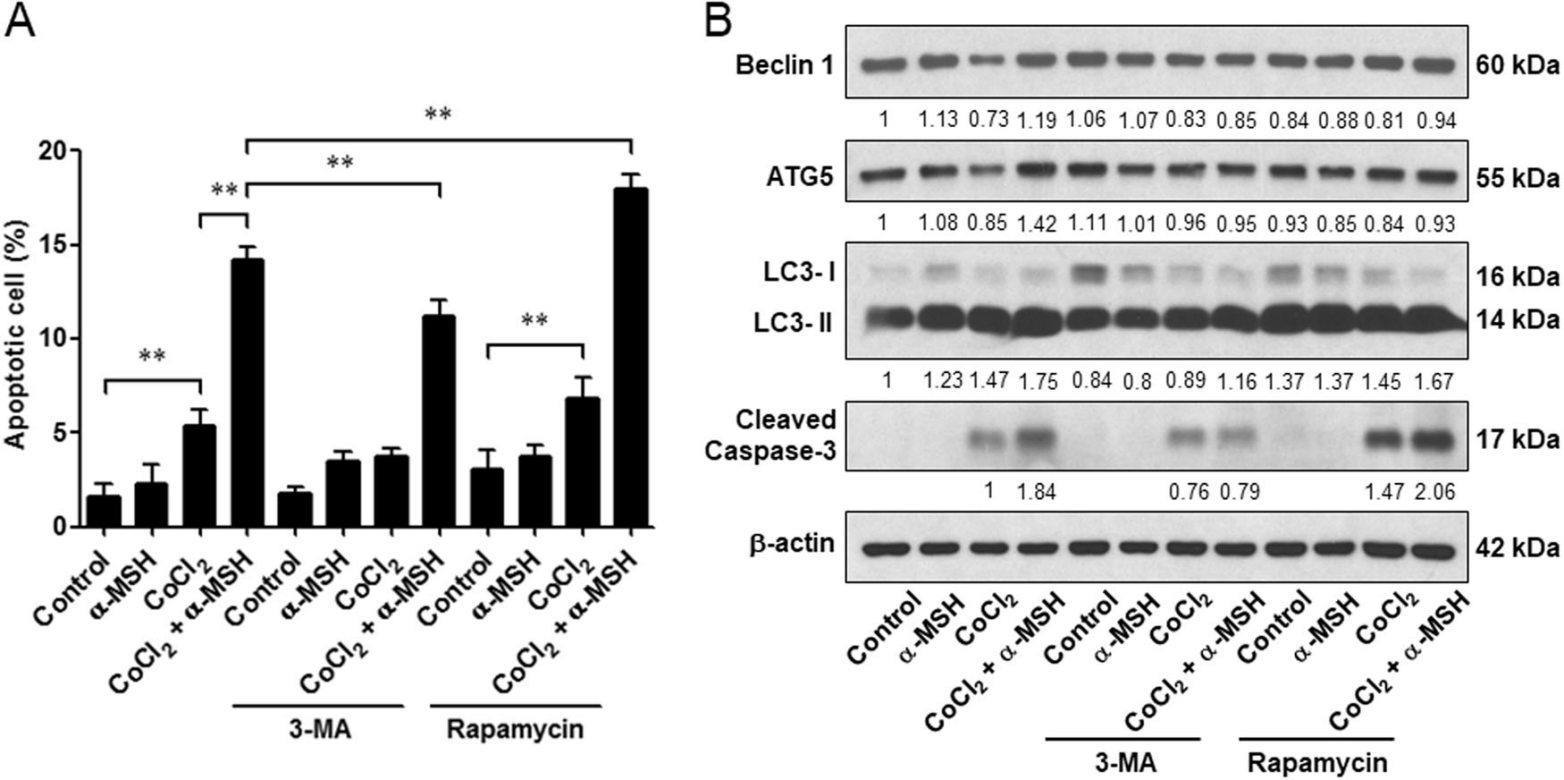
Effect of autophagy modulators on α-MSH-induced apoptosis in B16-F10 melanoma cells during hypoxia. After treatment with α-MSH for 24 h, cells were incubated with 3-MA or rapamycin in the presence of CoCl_2_ challenge for another 24 h. **a** Cell lysates were subjected to immunoblot with the indicated antibodies. β-Actin was used as an internal control for loading and transfer. **b** The population of apoptotic cells was analyzed by flow cytometry and qualified as mean ± SD from triplicate experiments. **p < 0.01. Autophagy inhibitor 3-MA decreased, whereas autophagy-inducer rapamycin increased, the α-MSH-induced apoptosis in B16-F10 melanoma cells during hypoxia

Because α-MSH simultaneously evokes autophagy and apoptosis in melanoma cells during hypoxia, it is pivotal to elucidate the role of autophagy on α-MSH-induced apoptosis in melanoma cells. By flow cytometric analysis, it was shown that application of autophagy inhibitor 3-MA potently attenuated the α-MSH-induced apoptosis in B16-F10 melanoma cells during hypoxia (from 14.19 to 11.23%). On the contrary, treatment with autophagy-inducer rapamycin significantly enhanced the α-MSH-induced apoptosis in melanoma cells (from 14.19 to 17.98%; Fig. 4a). Immunoblot analysis also revealed that 3-MA retarded α-MSH-induced Beclin 1, ATG5, and cleaved caspase-3 expression levels, while rapamycin was prominent to elevate the level of cleaved caspase-3 both in α-MSH treatment under hypoxic condition and CoCl_2_ only group (Fig. 4b).

### ATG5 silencing attenuated the α-MSH-induced apoptosis

**Fig. 5:**
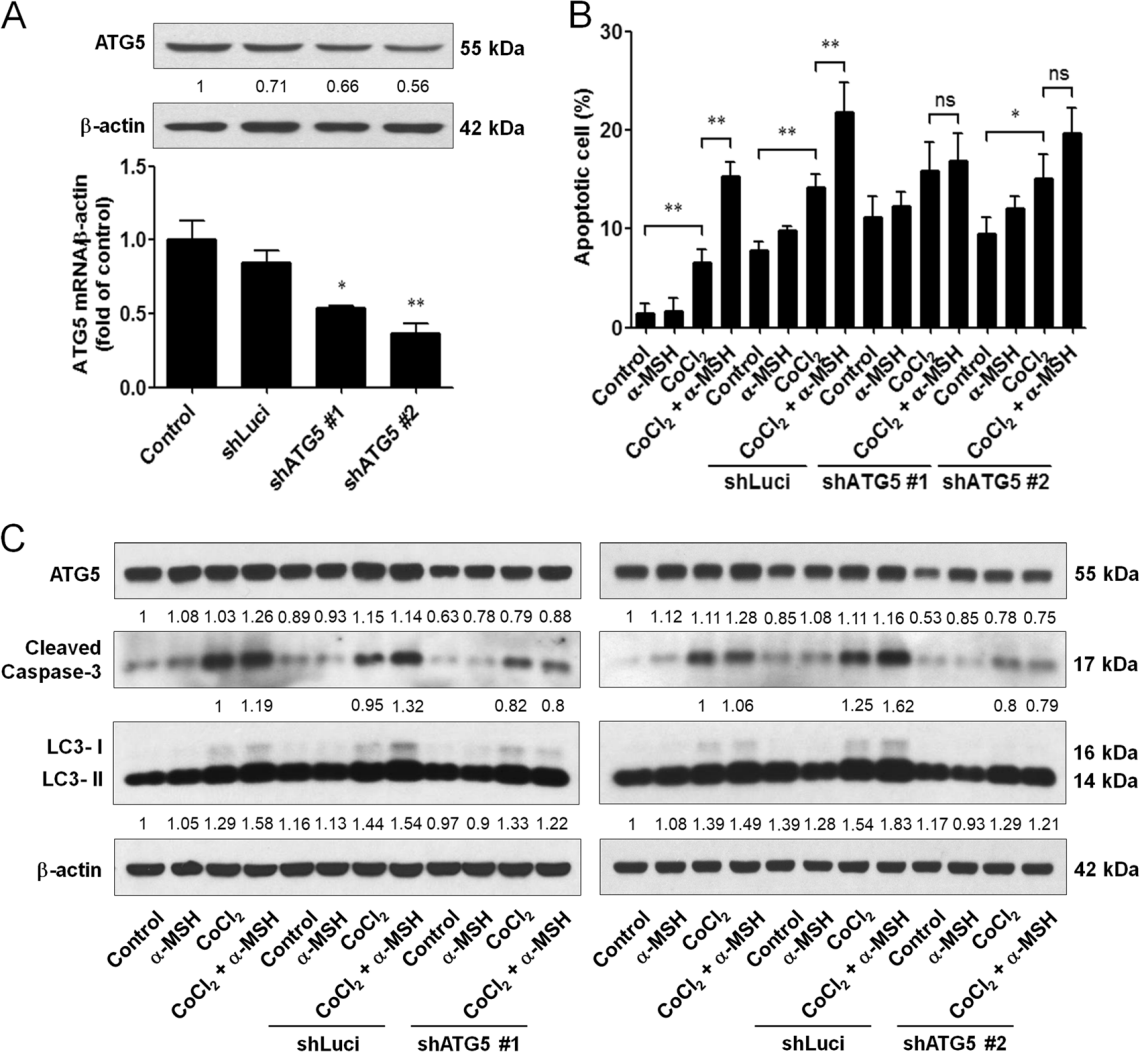
Effect of ATG5 knockdown on α-MSH-induced apoptosis in B16-F10 melanoma cells during hypoxia. **a** Cells were transfected with shLuci or shATG5 plasmids for 48 h before harvest. ATG5 shRNA reduced the basal ATG5 mRNA and protein expression levels. **b** Silencing ATG5 expression retarded α-MSH-induced apoptosis during hypoxia compared to the shLuci group. **c** Cell lysates were subjected to immunoblot with the indicated antibodies. β-Actin was used as an internal control for loading and transfer. *p < 0.05, **p < 0.01. Downregulation of ATG5 attenuated the α-MSH-induced apoptosis in B16-F10 melanoma cells during hypoxia

Since ATG5 is an important regulator of autophagy^30^, we investigated the effect of ATG5 knockdown on α-MSH-induced apoptosis. By RNA interference technique, it was demonstrated that shATG5 #1 and #2 plasmids' transfection were workable to the basal ATG5 mRNA and protein expression levels in B16-F10 melanoma cells (Fig. 5a). As expected, silencing ATG5 had a higher proportion of apoptosis in the control and CoCl_2_ groups, but no significant increase under α-MSH and hypoxia double stresses compared to CoCl_2_ only groups (Fig. 5b). Moreover, immunoblot analysis also revealed that silencing ATG5 was partially to diminish α-MSH-induced cleaved caspase-3 and LC3-ΙΙ expression levels during hypoxia (Fig. 5c). Therefore, these results proposed that α-MSH-induced autophagy was prone to boosting apoptosis rather than assist the cell survival during hypoxia.

### α-MSH augmented HIF-1α signaling to regulate Bcl-2 family gene expression levels in melanoma cells

**Fig. 6:**
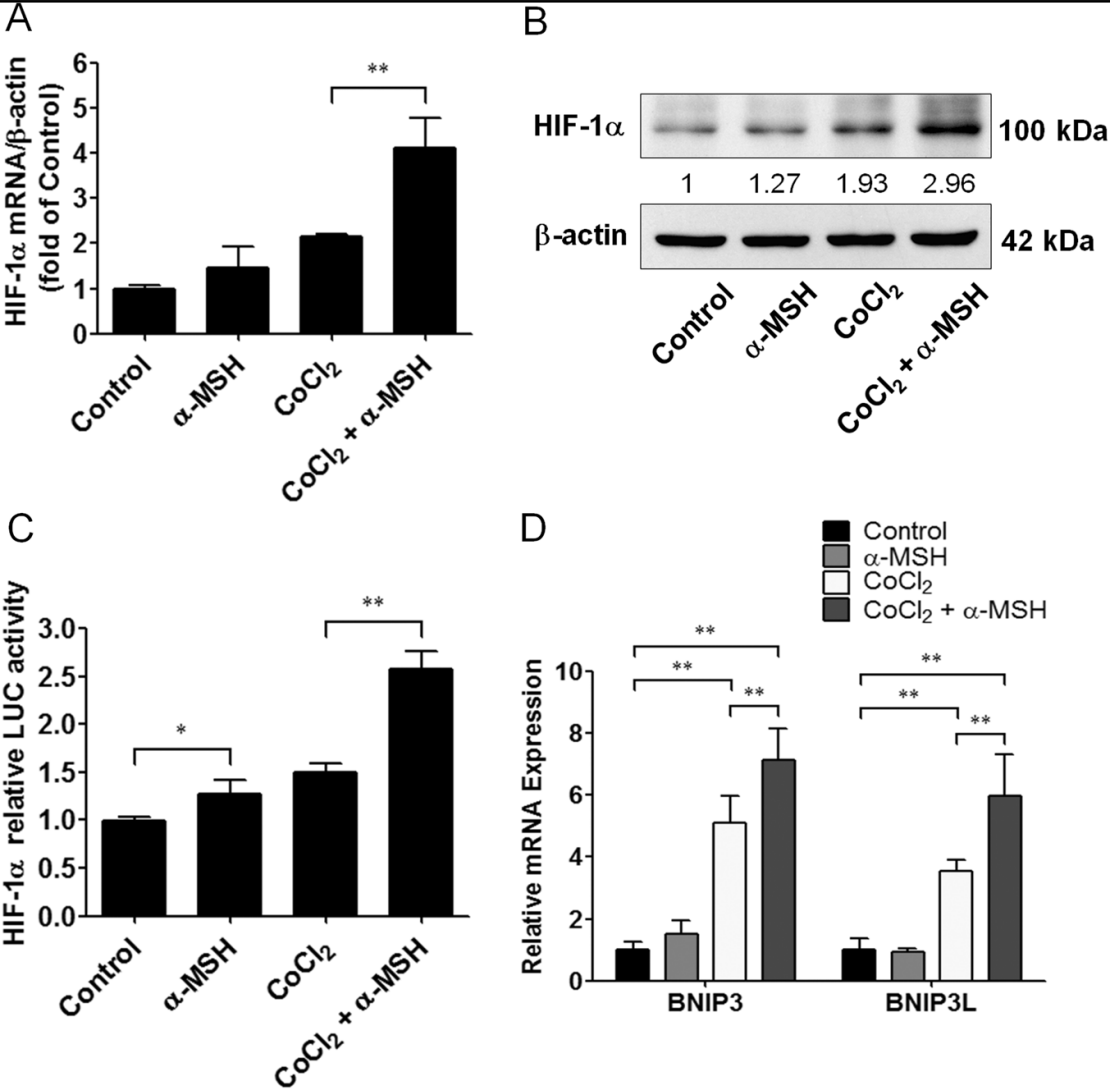
Effect of α-MSH on HIF-1α expression and activity in B16-F10 melanoma cells during hypoxia. **a**, **b** α-MSH increased HIF-1α mRNA and protein expression levels during hypoxia. **c** Cells were transfected with luciferase reporter driven by a promoter region containing HIF-1α-specific binding sites before α-MSH treatment at the indicated time and concentration. The luciferase activities were measured immediately and expressed as means ± SD from triplicate experiments. **d** Relative mRNA expression levels were analyzed by real-time PCR. Data are expressed as fold change compared with control (means ± SD of triplicate experiments). *p < 0.05, **p < 0.01. α-MSH elevated the Bcl-2 family gene expression levels by HIF-1α activation

Since HIF-1α/BNIP3/Beclin 1 is considered a general autophagy signaling pathway^31^, we thus investigated whether α-MSH-induced autophagy was via HIF-1α activation. By quantitative real-time PCR analysis, it was shown that α-MSH significantly augmented HIF-1α mRNA expression in B16-F10 melanoma cells during hypoxia compared with the CoCl_2_ only group, although the mRNA level of HIF-1α was only slight increased in α-MSH-treated cells in normoxia (Fig. 6a). Moreover, immunoblot and luciferase reporter assays have shown that α-MSH elevated the HIF-1α-driven luciferase activity and protein levels in both normoxia and hypoxia (Fig. 6b, c). The similar results also were obtained by POMC gene delivery in B16-F10 melanoma cells (Supplementary 1A-C). Because of the Bcl-2 family such as BNIP3 and BNIP3L were triggered by HIF-1α in autophagic process, we next evaluated whether α-MSH modulated the Bcl-2 family gene expression in melanoma cells during hypoxia. As expected, by quantitative real-time PCR analysis, it was demonstrated that α-MSH significantly increased the gene expression of BNIP3 and BNIP3L in B16-F10 melanoma cells exposed to hypoxic condition (Fig. 6d). Together, these results suggested that α-MSH-induced autophagy in hypoxia was via HIF-1α-mediated signaling pathway.

### HIF-1α silencing ameliorated the α-MSH-induced apoptosis in melanoma cells during hypoxia

**Fig. 7:**
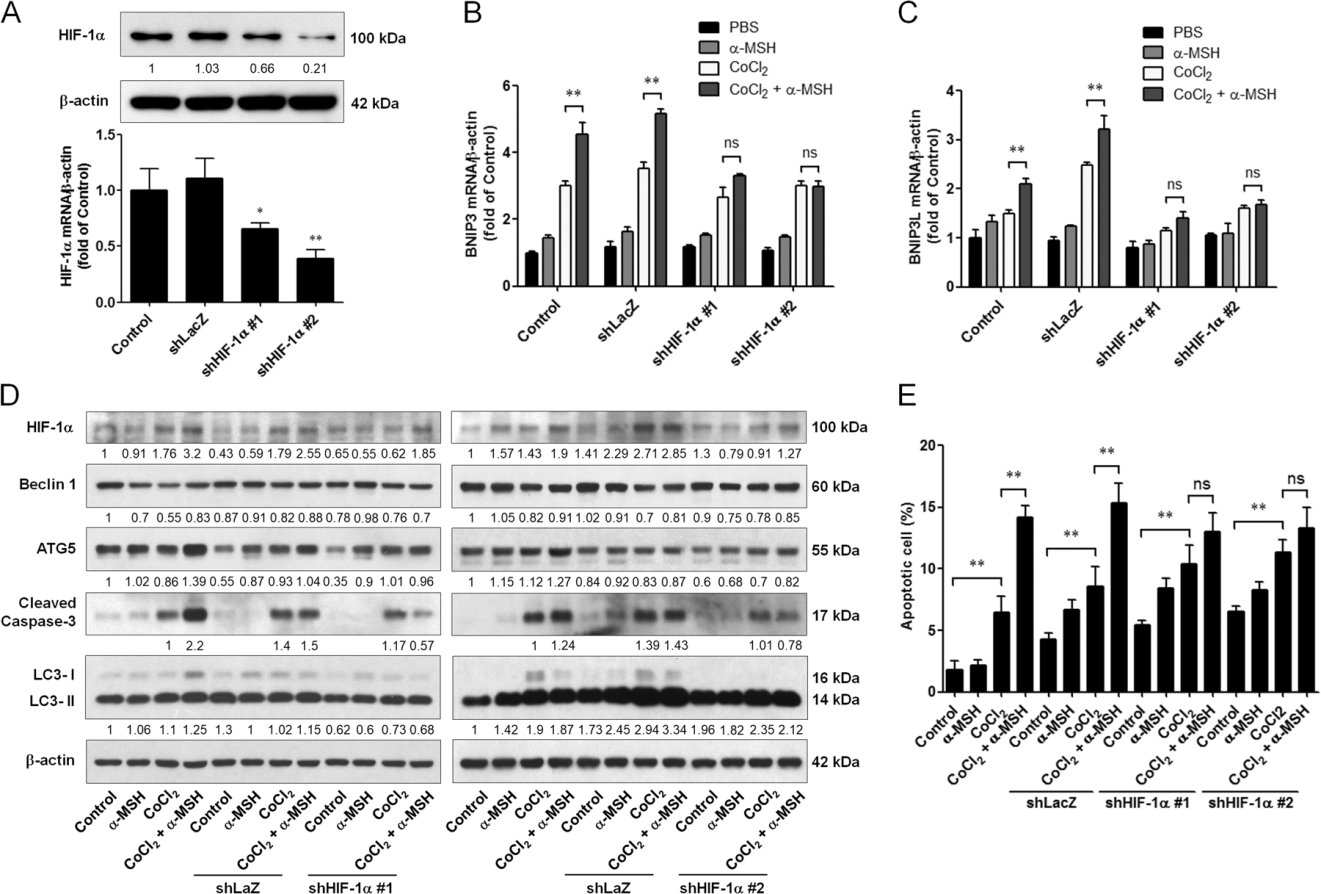
Effect of HIF-1α silencing on α-MSH-induced apoptosis in B16-F10 melanoma cells during hypoxia. **a** Cells were transfected with shLacZ or shHIF-1α plasmids for 48 h before harvest. HIF-1α shRNA reduced the basal HIF-1α mRNA and protein expression levels. **b**, **c** Relative mRNA expression levels were analyzed by real-time PCR. Data are expressed as fold change compared with control (means ± SD of triplicate experiments). **d** Cell lysates were analyzed by immunoblot using the indicated antibodies. β-Actin was used as an internal control for loading and transfer. **e** The population of apoptotic cells was analyzed by flow cytometry and qualified as mean ± SD from triplicate experiments. *p < 0.05, **p < 0.01. Knockdown of HIF-1α diminished α-MSH-induced apoptosis in B16-F10 melanoma cells during hypoxia

To investigate whether knockdown of HIF-1α could affect α-MSH-induced apoptosis in B16-F10 melanoma cells during hypoxia, HIF-1α short hairpin RNA (shRNA) plasmids were exploited to execute the following in vitro study analysis. By immunoblot and quantitative real-time PCR analysis, it was demonstrated that both of HIF-1α shRNA #1 and #2 transfection significantly attenuated the basal HIF-1α mRNA and protein expression levels (Fig. 7a). Moreover, knockdown of HIF-1α by shRNA was efficient to retard α-MSH-induced BNIP3 and BNIP3L gene expression during hypoxia (Fig. 7b, c). More importantly, α-MSH-induced LC3-ΙΙ and cleaved caspase-3 were partially abrogated in B16-F10 melanoma cells transfected with HIF-1α shRNA during hypoxia (Fig. 7d). Flow cytometric analysis also demonstrated that silencing HIF-1α did not elevate the proportion of α-MSH-induced apoptosis, contrarily no statistical difference was observed between α-MSH and CoCl_2_ double stresses and CoCl_2_ only groups (Fig. 7e). In summary, we herewith propose that α-MSH-induced autophagy partially contributed to cell apoptosis by HIF-1α/ BNIP3/BNIP3L signaling pathway.

## Discussion

This study has first demonstrated that POMC gene therapy simultaneously induces two different types of cell death: autophagy and apoptosis in melanoma in vivo. This was further validated by cell culture studies, in which POMC gene delivery elevated LC3-positive autophagosome formation in melanoma cells under hypoxia. We subsequently delineated that the POMC-derived α-MSH was sufficient to evoke the autophagy as well as apoptosis in melanoma cells in the presence of CoCl_2_. Interestingly, pharmaceutical intervention studies using autophagy modulators reveal that the α-MSH-evoked autophagy participates in the α-MSH-induced cell death rather than protection against environmental stress. This notion was later supported by ATG5 knockdown studies. Finally, we delineated that α-MSH augmented the HIF-1α signaling during hypoxia, which elevated the expression of downstream Bcl-2 family genes (including BNIP3 and BNIP3L) to elicit the activation of both autophagic and apoptotic cell death. Moreover, HIF-1α knockdown retarded the α-MSH-induced autophagosome formation and apoptosis in melanoma cells, implicating the pivotal role of HIF-1α activation in α-MSH-induced cell death. Therefore, the present study unveils that POMC therapy induces the activation of autophagic and apoptotic cell death pathways in melanoma cells through α-MSH/HIF-1α/BNIP3 signaling.

Autophagy is a self-consumption mechanism against adversities present within tumor microenvironment such as hypoxia and nutrition deprivation^6^. Here the present study proclaimed that α-MSH strikingly accelerated the LC3-ΙΙ expression and GFP-LC3 puncta spots under hypoxic stress. Interestingly, inhibition of autophagy by 3-MA or blocking ATG5 expression was enough to retard α-MSH-induced apoptosis, but autophagy-inducer rapamycin treatment contrarily increased the apoptosis of melanoma cells during hypoxia. Although autophagy is usually considered to protect tumor cells from apoptosis, these studies have implied that the induction of autophagy indeed participate in apoptotic process under some specific stress conditions^32–34^. Especially, ATG5 protein is indicated to directly interact with apoptosis-related protein and silencing ATG5 can reduce drug-induced cell death^35,36^. Therefore, our results suggested that the additional enhancement of autophagy contributed to α-MSH-induced cell death in B16-F10 melanoma cells during hypoxia.

Accumulation of ROS is considered as one of the important mediators of autophagy in response to several stress conditions^5^. Zhang and colleagues recently indicated that enhancement of autophagy by ROS stimuli accelerated cell apoptosis and growth inhibition of melanoma cells in vitro and in vivo^37^. Similar to this notion, the present study has shown that POMC-induced autophagy contributed to cell apoptosis in melanoma cells during hypoxia. In the meantime, we also found that mitochondrial morphology change and dysfunctions in Ad-POMC-infected or α-MSH-treated cells during hypoxia (data not shown). Additionally, our previous study has demonstrated that POMC-induced apoptosis was through ROS accumulation^29^. Therefore, we guessed that POMC-induced autophagy might directly regulate by ROS accumulation but this needs further investigation.

Given that HIF-1α upregulation is frequently implicated in response to hypoxic stress and autophagic process, the present study showed that α-MSH significantly increased HIF-1α transcriptional activity and both protein and mRNA levels in B16-F10 melanoma cells during hypoxia, and HIF-1α-mediated Bcl-2 family genes (BNIP3 and BNIP3L) were also elevated by double stresses (α-MSH and hypoxia). This finding is consistent with previous report suggesting that hypoxia-induced autophagy is through HIF-1α-mediated autophagy genes' (BNIP3 and BNIP3L) expression^38^. Moreover, our results further demonstrated that silencing HIF-1α by RNA interference abolished the α-MSH-stimulated autophagy genes' expression and eventually retarded α-MSH-induced apoptosis in melanoma cells during hypoxia. Therefore, we hypothesize that the induction of autophagy genes by HIF-1α could contribute to α-MSH-induced apoptosis in B16-F10 melanoma cells during hypoxia as previously described^32,39^.

In summary, the present study unveils that POMC gene therapy suppresses melanoma through activation of both autophagic and apoptotic signaling pathways in melanoma cells. Besides, combination therapy using α-MSH in conjunction with autophagy inducer such as rapamycin may facilitate a novel therapeutic strategy for melanoma control.

## Materials and Methods

### Cell cultures and reagents

Human MNT-1 melanoma cells were acquired as gift from Dr. Chung-Hsing Chang (Kaohsiung Medical University, Taiwan). Mouse (B16-F10) and human (A375 and A2058) melanoma cells were purchased from American Type Culture Collection (Manassas, VA, USA). These cells were cultured in Dulbecco's modified Eagle’s medium (Invitrogen; Carlsbad, CA, USA) containing 10% fetal bovine serum (Hyclone, Logan, UT, USA), 2 mM glutamine, 100 mg/ml streptomycin (Invitrogen; Carlsbad, CA, USA), and 100 U/ml penicillin at 37 °C in 5% CO_2_ atmosphere. The following reagents were purchased from Sigma-Aldrich (St. Louis, MO, USA): 3-MA (M9281), rapamycin (R0395), CoCl_2_ (60818), and β-actin (A5441). The following antibodies were purchased from Cell Signaling Technology (Danvers, MA, USA): Beclin1 (3738), LC3A/B (12741), ATG5 (12994), and Caspase-3 (9662). Antibody against HIF-1α (NB100-479) was purchased from Novus (St Louis, MO, USA). Other antibodies were purchased from Santa Cruz Biotechnology (Santa Cruz, CA, USA): Bax (sc-7480) and Bcl-2 (sc-7382). The α-MSH peptide was purchased from BACHEM (Torrance, CA, USA).

### Preparation of Ad vectors

Preparation of recombinant Ads containing POMC gene (Ad-POMC) or control GFP (Ad-GFP) were performed as previously described^24^.

### Flow cytometric analysis

The apoptosis of B16-F10 cells after gene delivery or α-MSH treatment was analyzed by flow cytometry as described previously^29^. Briefly, cell aliquots were incubated with RNase A (10 µg/ml) and propidium iodide (50 µg/ml) for 30 min at 37 °C. DNA content of 10,000 events was analyzed using a FACS Caliber flow cytometer (Becton Dickinson Biosciences; San Jose, CA, USA) and the CELLQuest software.

### Immunofluorescence staining of fixed tumor sections

After deparaffinization, the slides were blocked with 3% hydrogen peroxide for 10 min and subjected to antigen retrieval with microwave in 10 mM citrate buffer for 15 min. Then subsequently, the slides were blocked with a 5% (v/v) bovine serum albumin (BSA) and 0.1% (v/v) Triton X100 for 30 min and incubated with the indicated primary antibodies at 4 °C overnight. After a wash with phosphate buffer solution, the sections were incubated with Alexa Fluor-conjugated secondary antibody (Invitrogen; Carlsbad, CA, USA) for 1 h and subsequently counterstained with 4,6-diamidino-2-phenylindole for 10 min and viewed under a fluorescent microscope.

### Confocal microscopy

Cells were grown on glass coverslips for 16 h. After gene delivery or peptide treatment, cells were stained with MitoTracker Orange CM-H_2_TMRos (Molecular Probes Inc., M7511) for 15 min at 37 °C and then cells were fixed with 4% (v/v) paraformaldehyde for 5 min and permeabilized with phosphate-buffered saline containing 0.1% (v/v) Triton X-100 and 2% (v/v) BSA at room temperature for 5 min. Cells were then labeled with anti-Bax (Santa Cruz Inc; Santa Cruz, CA, USA) antibody for 30 min and with Alexa Fluor-conjugated goat anti-mouse antibody (Invitrogen; Carlsbad, CA, USA) for 30 min. Labeled cells were visualized with LSM510 (Carl Zeiss, Thornwood, NY, USA).

### Autophagy analysis by GFP-LC3

Cells grown on glass coverslips were transfected with GFP-LC3 plasmid DNA (a gift from Dr. T. Yoshimori, Osaka University, Osaka, Japan) using Lipofectamine 3000 Reagent (Invitrogen; Carlsbad, CA, USA) based on the manufacturer’s instructions for 24 h^40^. After transfection, cells were incubated with α-MSH (10 nM) in the presence or absence of CoCl_2_ (100 μM) for another 24 h. The formation of GFP-LC3 punctate structures were visualized with LSM510 (Carl Zeiss, Thornwood, NY, USA).

### Gene knockdown using shRNA

Various pLKO.1 plasmids to knockdown HIF-1α, ATG5, and scramble control were purchased from National RNAi Core Facility of Academia Sinica (Taipei, Taiwan). Briefly, cells were transiently transfected with 2 μg of plasmids by Lipofectamine 3000 Reagent (Invitrogen; Carlsbad, CA, USA) according to manufacturer’s instructions. After transfection for 24 h, cells were treated with α-MSH (10 nM) for 24 h and then incubated in the presence or absence of CoCl_2_ (100 μM) for another 24 h before harvest for the indicated assays.

### HIF-1α luciferase assay

Cells were co-transfected with HIF-1α-driven luciferase (Stratagene, La Jolla, CA, USA) vector and the *Renilla reniformis* luciferase reporter vector (Promega, Madison, WI, USA) at a ratio of 1:1/10 using Lipofectamine 3000 Reagent (Invitrogen; Carlsbad, CA) for 4 h before being maintained with fresh medium for 24 h. Subsequently, cells were treated with α-MSH (10 nM) for 24 h and then incubated in the presence or absence of CoCl_2_ (100 μM) for another 24 h. The HIF-1α-driven luciferase activities in cells were measured using a Dual-Light Kit (Promega, Madison, WI, USA) in Orion II microplate luminometer (Titertek Berthold; Pforzheim, Germany) and normalized with that of *R. reniformis* luciferase according to the manufacturer’s instructions.

### Western blot analysis

Cell lysates were prepared and the level of protein expression was measured as previously described^27^. The  PVDF membrane was blocked with 5% milk in TBS-T for 1 h and then incubated with specific primary antibodies and secondary antibodies conjugated with horseradish peroxidase (HRP; 1:10,000 dilutions in 5% milk) for 1 h, respectively. The signals on membrane were detected using chemiluminescent HRP substrate (Millipore Corporation; Billerica, MA, USA) and exposed to X-ray film for autoradiogram.

### Quantitative real-time PCR

RNA was purified and quantitative real-time PCR was performed as previously described^29^. The cDNA product was used for quantitative real-time PCR by the SYBR Green PCR master mix and the predesigned gene-specific probe and primer sets for mouse HIF-1α (NM_010431.2), BNIP3 (NM_009760.4), and BNIP3L (NM_009761.3). Data were normalized to β-actin (NM_007393.3) and expressed as fold changes over that in the control experiments. The primer sequences were as follows: HIF-1α forward primer (5′-ATG TGA CCA TGA GGA AAT GAG AGA A-3′) and reverse primer (5′-CTG AGG TTG GTT ACT GTT GGT ATC A-3′); BNIP3 forward primer (5′-ACA CCA CAA GAT ACC AAC AGA G-3′) and reverse primer (5′-**TGT TTC TCA TGC TGA GAG TAG C**-3′); BNIP3L forward primer (5′-GCC CTT CAC CAC AAG AAG AT-3′) and reverse primer (5′-**TTA GAG ACG CAG CAC GTT TAG**-3′); and β-actin forward primer (5′-GGA ATC CTG TGG CAT CCA T-3′) and reverse primer (5′-**GCT CAG GAG GAG CAA TGA T** -3′).

### Statistical analysis

All data are expressed as the mean ± standard deviation (SD). Statistical analysis was performed with one-way analysis of variance followed by Newman–Keuls post hoc or *t* test (for multiple comparisons) using Prism ver.5 (GraphPad Software, Inc., California, USA). A *P* value of <0.05 was regarded as statistically significant.

## Electronic supplementary material

Supplementary Figure 1. Effect of POMC gene delivery on HIF-1α expression and activity in B16-F10 melanoma cells during hypoxia
