# Supplementary figures and images for: Autophagic cell death participates in POMC-induced melanoma suppression
Source: Cell Death Discov. 2018 Jul 10;4:68. doi: 10.1038/s41420-018-0070-5 (PMC6060113; doi:10.1038/s41420-018-0070-5)

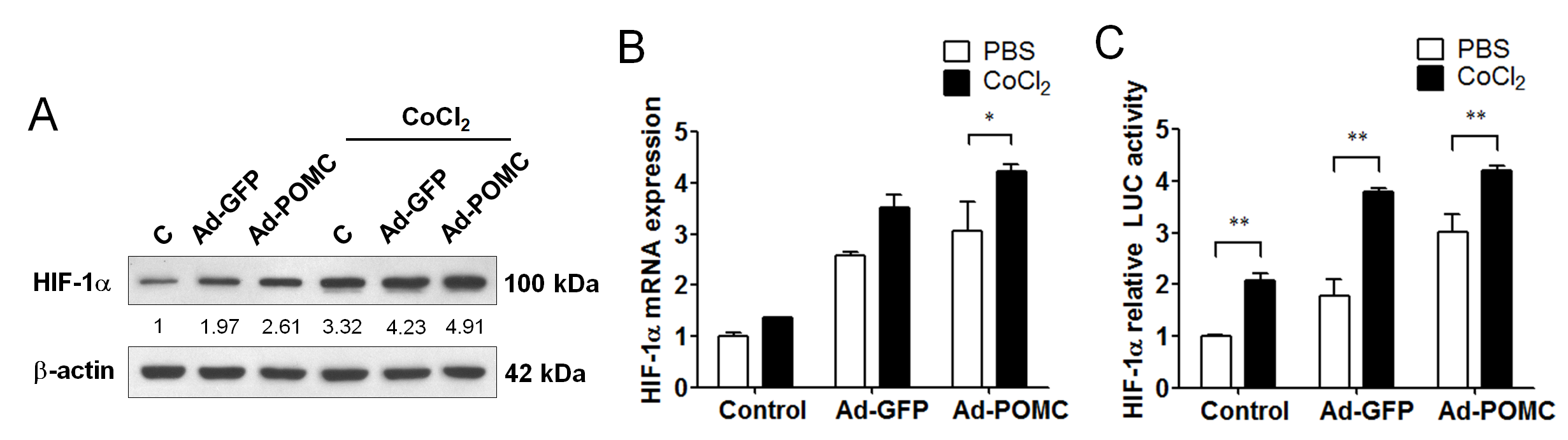

Supplement: Supplementary file 1 — Supplementary Figure 1. Effect of POMC gene delivery on HIF-1α expression and activity in B16-F10 melanoma cells during hypoxia [file 41420_2018_70_MOESM1_ESM.tif]
